# The reticular lamina and basilar membrane vibrations in the transverse direction in the basal turn of the living gerbil cochlea

**DOI:** 10.1038/s41598-022-24394-0

**Published:** 2022-11-17

**Authors:** Wenxuan He, George Burwood, Edward V. Porsov, Anders Fridberger, Alfred L. Nuttall, Tianying Ren

**Affiliations:** 1 Department of Otolaryngology, Oregon Hearing Research Center, Oregon Health and Science University, Portland, OR 97239 USA; 2 Department of Biomedical and Clinical Sciences, Linköping University, 581 83 Linköping, Sweden

**Keywords:** Neuroscience, Cochlea, Hair cell, Inner ear, Transduction

## Abstract

The prevailing theory of cochlear function states that outer hair cells amplify sound-induced vibration to improve hearing sensitivity and frequency specificity. Recent micromechanical measurements in the basal turn of gerbil cochleae through the round window have demonstrated that the reticular lamina vibration lags the basilar membrane vibration, and it is physiologically vulnerable not only at the best frequency but also at the low frequencies. These results suggest that outer hair cells from a broad cochlear region enhance hearing sensitivity through a global hydromechanical mechanism. However, the time difference between the reticular lamina and basilar membrane vibration has been thought to result from a systematic measurement error caused by the optical axis non-perpendicular to the cochlear partition. To address this concern, we measured the reticular lamina and basilar membrane vibrations in the transverse direction through an opening in the cochlear lateral wall in this study. Present results show that the phase difference between the reticular lamina and basilar membrane vibration decreases with frequency by ~ 180 degrees from low frequencies to the best frequency, consistent with those measured through the round window. Together with the round-window measurement, the low-coherence interferometry through the cochlear lateral wall demonstrates that the time difference between the reticular lamina and basilar membrane vibration results from the cochlear active processing rather than a measurement error.

## Introduction

The high spatial resolution of low-coherence interferometry, including heterodyne^1,2^ and homodyne^3^ low-coherence interferometry and optical coherence tomography^4–11^, allows auditory scientists to measure sub-nanometer vibrations within the cochlear partition in living cochleae. Pioneering application of homodyne low-coherence interferometry demonstrated the differential motions between the reticular lamina (RL) and basilar membrane (BM) in living guinea pig cochleae^12^. The deep penetration of infrared light makes it feasible to measure sound-induced vibrations through the thin bony cochlear shell at the apex or upper cochlear turns^5,6,9,10,13–16^. However, the thick bony walls of the cochlear basal turn attenuate the incident and backscattered light dramatically, making it difficult to measure the vibrations in the transverse direction through the intact bony walls. Since the cochlear basal turn is responsible for a large portion of audible frequencies due to the logarithmic scale of the cochlear frequency-location map^17^, the cochlear partition vibrations in the basal turn have been measured through the round window with^2,7,8,18–21^ or without^22–24^ an intact round window membrane. This approach has been used in micromechanical measurements in mice, gerbils, and guinea pigs in recent years. Similar to guinea pig data^25^, the magnitude responses measured from the sensitive mouse and gerbil cochleae show that the RL vibrates significantly more than the BM at the best frequency and low frequencies. Phase responses indicate that the RL group delay is significantly larger than that of the BM. RL and BM move approximately in opposite directions at low frequencies and in the same direction at the best frequency^22–24,26^.

**Figure 1:**
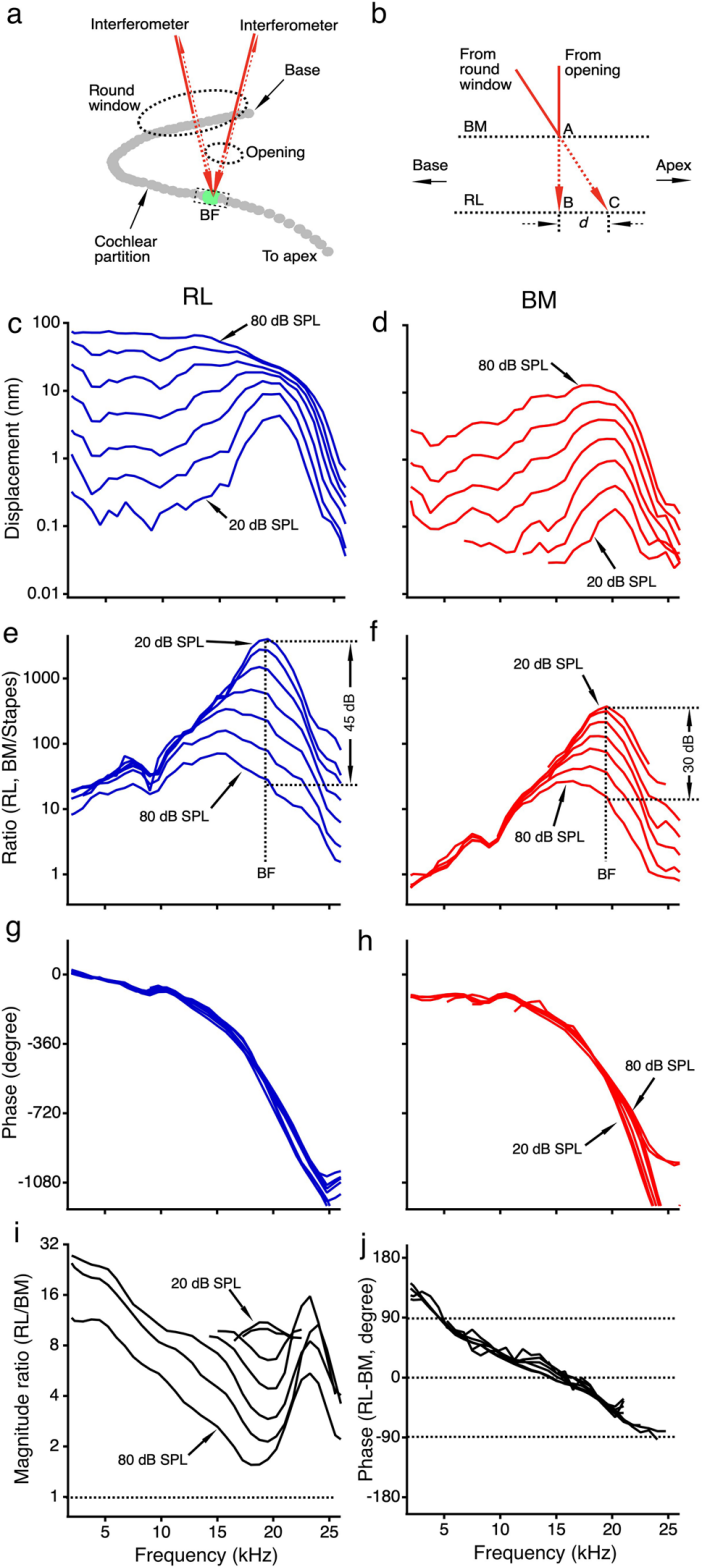
Schematic of optical accesses to the cochlear partition and vibrations of the reticular lamina and basilar membrane. (**a**) While the object beam of the interferometer accesses the cochlear partition through the round window in a non-perpendicular direction, it approaches the same best-frequency (BF) location through an opening in the cochlear lateral wall approximately in the transverse direction. (**b**) With a perpendicular optical axis, the object beam from the lateral wall opening reaches the BM and RL at the same longitudinal location (indicated by A and B in Fig. 1b). With a non-perpendicular optical axis, the object beam from the round window reaches the BM and RL at different longitudinal locations indicated by locations A and C. The time for a traveling wave to propagate over the distance between locations B and C can result in a phase difference between the RL and BM vibration. (**c**,**d**), The magnitude spectra of the RL and BM vibrations presented by displacements as a function of frequency at different sound pressure levels. (**e**,**f**) Transfer functions of the RL and BM vibrations presented by the ratio of the RL or BM displacement to the stapes displacement as a function of frequency at different sound pressure levels. (**g**,**h**) The phase spectra of the RL and BM vibrations shown by phase difference obtained by subtracting the stapes phase from the RL or BM phase as a function of frequency at different sound pressure levels. (**i**) Magnitude difference between the RL and BM vibration displayed by the ratio of the RL displacement to the BM displacement as a function of frequency at different sound pressure levels. (**j**) The phase difference between the RL and BM vibration obtained by subtracting the BM phase from the RL phase as a function of frequency at different sound pressure.

Due to the spatial relationship between the round window and the cochlear partition, the object beam of the interferometers may not be able to access the cochlear partition in the transverse direction through the round window (Fig. 1a). Under such a condition, the object beam might pass the BM and RL at different longitudinal locations (indicated by locations A and C in Fig. 1b). Distance between locations B and C can result in a phase difference between the RL and BM vibration. This possible systematic measurement error might account for the observed phase difference between the RL and BM vibration^27^.

This study aims to determine whether the systematic error caused by the non-perpendicular optical axis is responsible for the observed phase difference between the RL and BM vibration. The working hypothesis is that the measurement error causes the phase difference between the RL and BM. This hypothesis was tested by measuring RL and BM vibrations in the transverse direction through an opening in the bony lateral wall of the basal cochlear turn. It was expected that the data collected using this new approach would show no significant phase difference between the RL and BM vibration because the object beam could access the cochlear partition in the transverse direction. The present result rejects the hypothesis and confirms that the magnitude and phase differences between the RL and BM vibration^22,24^ are not caused by the non-perpendicular optical axis. Since the current method essentially eliminates the measurement angle-induced error, it can be used as an alternative approach for micromechanical measurement or imaging the organ of Corti in vivo at the basal cochlear turn in gerbils.

## Results

All gerbils survived surgeries, and there was no severe bleeding when the stapedial artery was removed. Although great care was taken to preserve the cochlear sensitivity, there was a > 6-dB decrease in the distortion product otoacoustic emission in six animals. The results presented below are from five of nineteen animals. Data from other preparations are excluded because of poor signal-to-noise ratio of interferometry.

### The reticular lamina and basilar membrane vibrations in the transverse direction

The displacement magnitude and phase of single tone-induced RL and BM vibrations were measured as a function of frequency in a sensitive cochlea and presented in Fig. 1c, d, g, and h. At 20 dB SPL (0 dB SPL = 20 µPa), the displacement of the RL vibration increases with frequency reaching the maximum at ~ 19.5 kHz, the best frequency of the measured cochlear location, and then decreases above this frequency (Fig. 1c). While the displacement increases proportionally with the sound pressure level up to 70 dB SPL at frequencies below 10 kHz, it increases at a much slower rate at the best frequency. For a 60-dB or a thousand-fold increase in the sound pressure, the RL displacement increases by < 20 dB or < ten-fold at ~ 19.5 kHz. Due to this frequency-dependent increase, the pattern of the RL magnitude response changes with the sound pressure level. At low and intermediate sound levels, the RL magnitude responses show a sharp peak centered at the best frequency. The peak expands toward low frequencies more than toward the high frequencies as the sound pressure level increases. At high sound pressure levels, the RL responses show a low-pass pattern indicating that a broad region of the cochlear partition from the base to the best-frequency location is excited.

The BM displacement is more than 20-dB or ten-fold smaller than that of the RL, not only at the best frequency but also at frequencies far below the best frequency at sound levels below 40 dB SPL (Fig. 1d). The BM displacement increases proportionally with the stimulus level at all sound pressure levels at frequencies below 10 kHz but at a slower rate at the best frequency. For a 60-dB sound pressure increase, the BM displacement at the best frequency increases by ~ 30 dB, which is more than that of the RL displacement.

Transfer functions of the RL and BM vibrations are presented by the ratio of the RL and BM displacement to the stapes displacement as a function of frequency in Fig. 1e and f. The RL transfer functions at low sound pressure levels show a sharp peak at ~ 19.5 kHz. The peak magnitude is > 1,000 and decreases by ~ 45 dB or ~ 200-fold as the sound pressure level increases from 20 to 80 dB SPL. The magnitude at frequencies below 10 kHz is independent of the sound pressure level up to 70 dB SPL. The magnitudes of the BM transfer function at and below the best frequency are more than 20-dB smaller than those of the RL transfer function. Compared to the ~ 45-dB decrease of the RL, the peak magnitude of the BM transfer function decreases by ~ 30 dB as the sound pressure level increases by 60 dB (Fig. 1f).

The phase spectra of the RL and BM vibrations are presented in Fig. 1g and h. The phase value was obtained by subtracting the stapes phase from the measured RL or BM phase. Both RL and BM phases decrease with frequency at an increasing rate. Given the constant distance from the stapes to the best-frequency location, the phase data indicate that the propagation speed of the vibration decreases with frequency, an indicator of the dispersion of the cochlear traveling wave. While there is no evident phase change with the sound pressure level at and below the best frequency, the phase increases slightly with the sound pressure level at frequencies above the best frequency. At frequencies below 15 kHz, the RL phase leads the BM phase, and the RL phase slope is steeper than that of the BM phase at low frequencies.

### The magnitude and phase differences between the reticular lamina and basilar membrane vibration

The magnitude relationship between the RL and BM vibration is presented by the ratio of the RL displacement to the BM displacement as a function of frequency in Fig. 1i. At 20 dB SPL, the displacement ratio is > 10 at the best frequency. This ratio decreases with the sound pressure level and becomes < 2 at 80 dB SPL. The displacement ratio also varies with frequency and decreases as the frequency increases reaching the minimum at the best frequency at intermediate and high sound pressure levels. In contrast to the RL and BM transfer functions in Fig. 1e and f, the largest displacement ratio of the RL to BM is at the low frequencies. The data in Fig. 1i indicates that the RL vibrates more than the BM at all frequencies and sound pressure levels. The decrease of the displacement ratio at the best frequency with the sound pressure level suggests that the nonlinear compression of the RL is stronger than that of the BM. Moreover, the pattern of the displacement ratio curves in Fig. 1i reveals that the largest amplitude difference between the RL and BM vibration is at low frequencies rather than at the best frequency.

The time relationship between the RL and BM vibration is presented by the phase difference as a function of frequency in Fig. 1j. The phase difference was obtained by subtracting the BM phase from the RL phase at different frequencies. At low frequencies, the phase difference is > 90 degrees, decreasing with frequency by ~ 180 degrees at the best frequency. There is no significant level-dependent change in the phase difference. The slope of the phase difference curves suggests a group delay difference between the RL and BM vibration. The slope of the phase difference curve at 70 dB SPL reveals a latency of ~ 26 μs.

**Figure 2:**
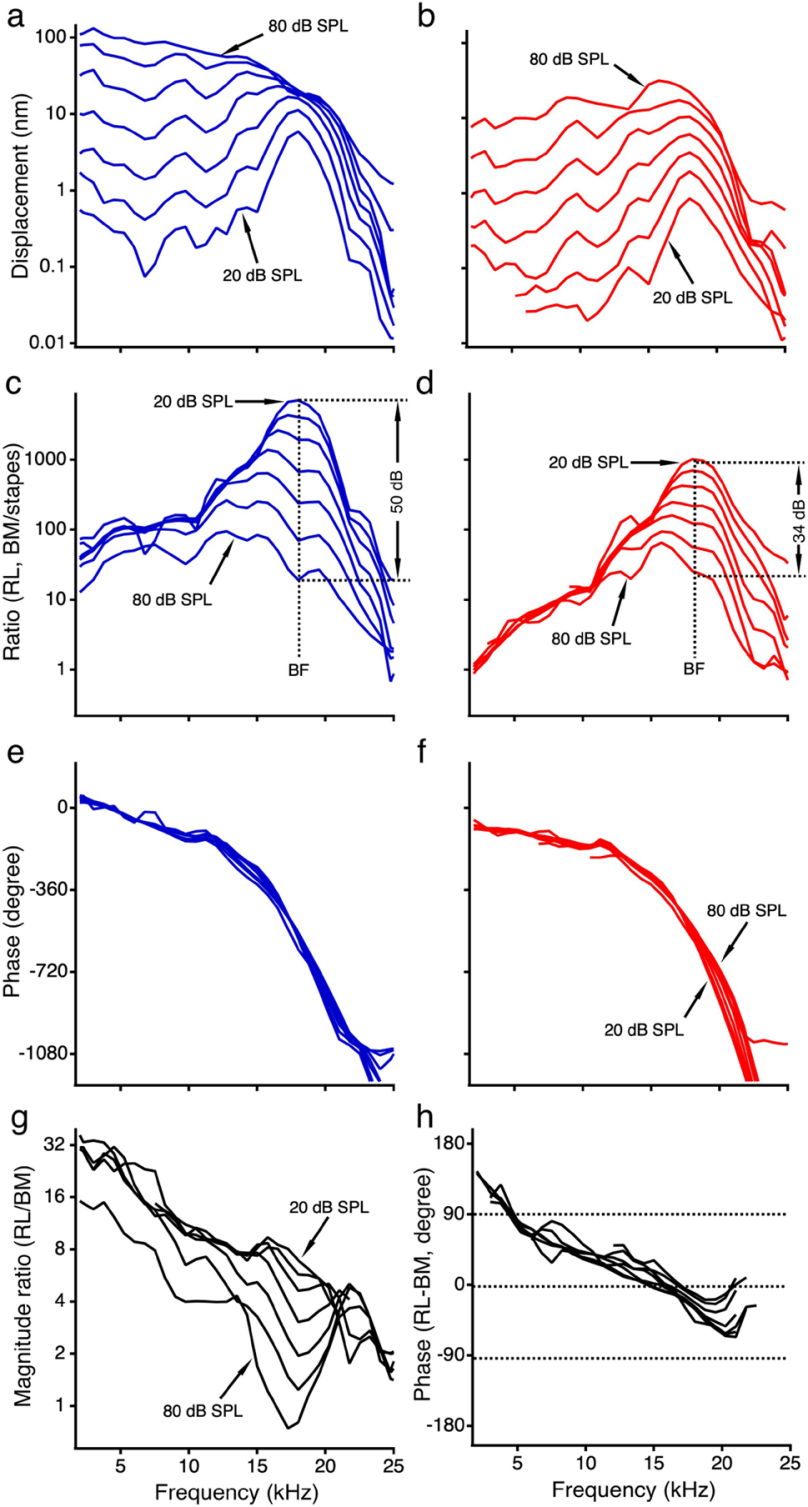
The reticular lamina and basilar membrane vibrations measured from a different sensitive cochlea. The RL and BM magnitude (**a**,**b**) and phase (**e**,**f**) responses, magnitude transfer functions (**c**,**d**), and the magnitude and phase differences between the RL and BM vibration (**g**,**h**) are similar to panels (**c**–**j**) in Fig. 1.

Another data set from a different sensitive cochlea is presented in Fig. 2. The similarity between Fig. 1c–j and Fig. 2 demonstrates the reproducibility of the results. The nonlinear compression, sharp tuning, and the difference between the RL and BM vibration were found in all sensitive preparations. The nonlinear compressive growth and sharp frequency tuning of the BM vibration measured in the transverse direction in this study are consistent with previous reports^22,28–30^.

**Figure 3:**
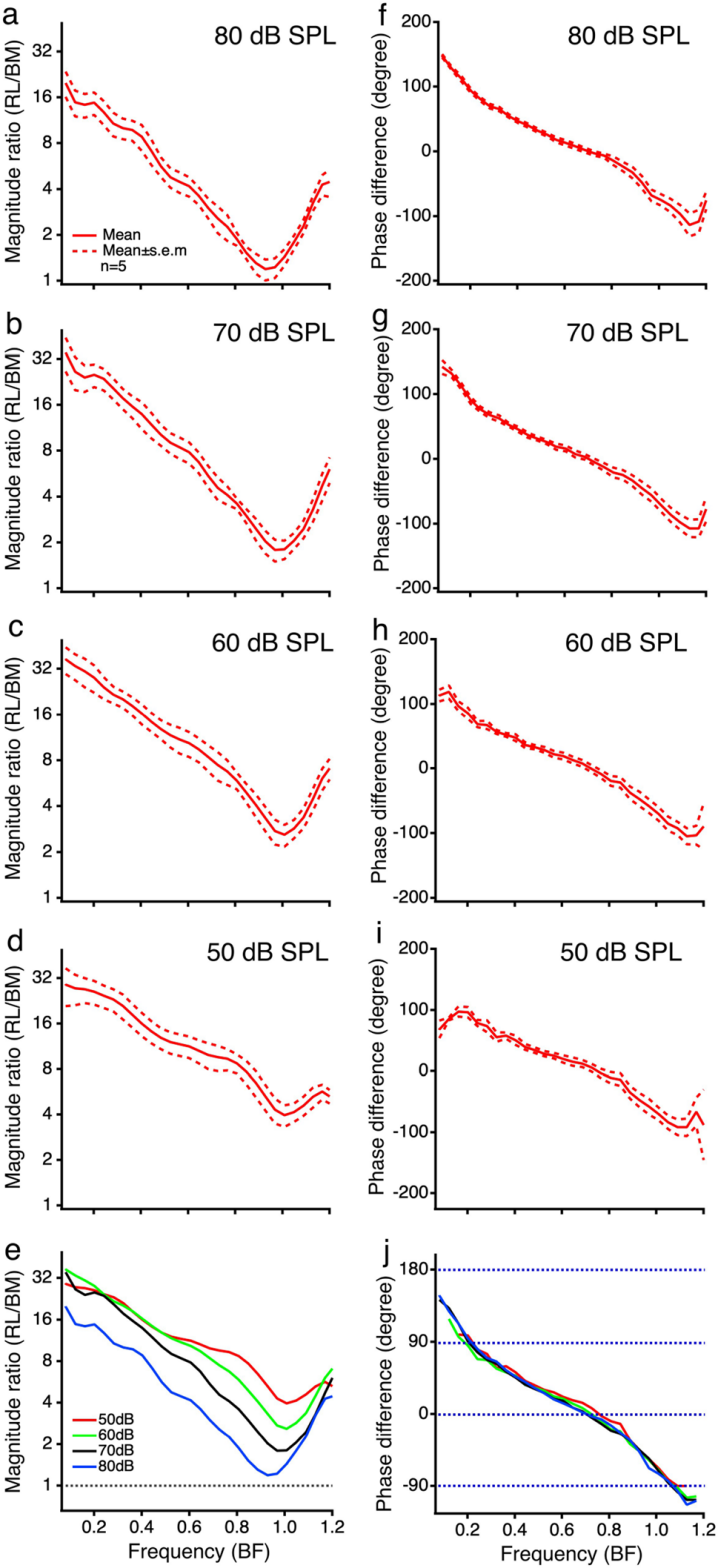
The magnitude and phase differences between the reticular lamina and basilar membrane vibration in five sensitive cochleae. (**a**–**d**), Means and standard errors of the magnitude difference between the RL and BM vibration presented by the ratios of the RL displacement to the BM displacement as a function of frequency at 80, 70, 60, and 50 dB SPL. Displacement ratios are the largest at low frequencies below 0.2 BF and decrease and then increase with the frequency reaching a minimum at the BF. All magnitude ratios are larger than one, indicating that the RL vibrates more than the BM. (**f**–**i**), Means and standard errors of the phase difference between the RL and BM vibration as a function of frequency at different sound pressure levels. The phase difference decreases with frequency at a similar rate at all sound pressure levels. (**e**), Means of the magnitude differences between the RL and BM vibrations at different sound levels. The magnitude difference curves shift downward as the sound pressure increases, suggesting the strongest nonlinear compression near BF. (**j**), Means of the phase differences between the RL and BM vibration at different sound pressure levels. Overlapping phase difference curves indicate that the group delay difference between the RL and BM vibrations does not change with the sound pressure level.

The magnitude and phase differences between the RL and BM vibration measured in five sensitive cochleae are presented as a function of frequency in Fig. 3. For statistical analysis, frequency axes were normalized to the best frequency for each animal. The magnitude difference between the RL and BM vibration is displayed by the ratio of the RL displacement to BM displacement in Fig. 3a–d. At all sound pressure levels, the displacement ratio is > 20 at frequencies of 0.1 BF, decreasing with frequency reaching the minimal at the BF. At frequencies above the BF, the magnitude ratio increases with frequency again. All magnitude ratios larger than one (black dotted line in Fig. 3e) indicate that the RL vibrates more than the BM at all frequencies and sound pressure levels. As the sound level increases, the magnitude ratio curve moves downward, indicating a compressive nonlinearity. The large separation between the curves at the BF suggests the strongest compression at this frequency. The temporal relationship between the RL and BM vibration is revealed by phase difference in Figs. 3f–i. The phase difference is > 90 degrees at frequencies below 0.1 BF and decreases with frequency at all sound pressure levels. The patterns of phase curves are similar across different sound pressure levels (Fig. 3j).

### Group delay difference between the reticular lamina and basilar membrane vibration

**Figure 4:**
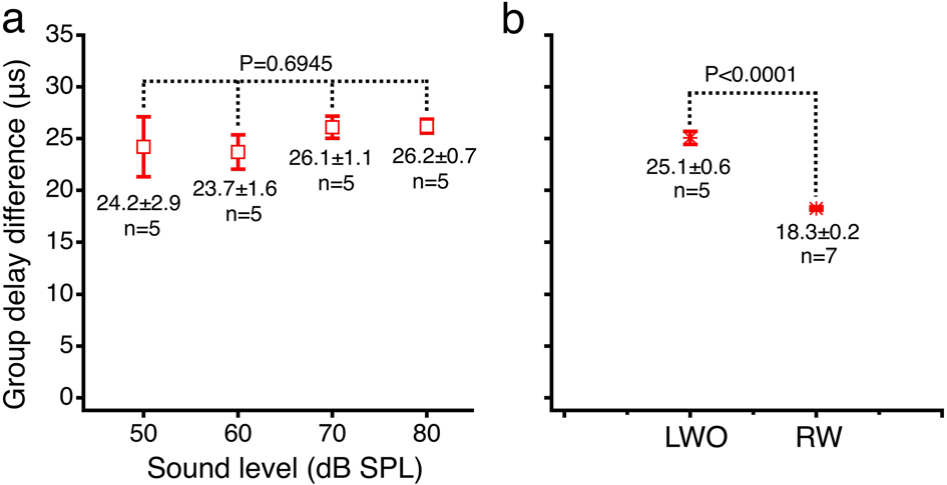
Group delay differences between the reticular lamina and basilar membrane vibration. (**a**), The group delay differences between the RL and BM vibration at different sound pressure levels. A one-way ANOVA showed that there was no statistically significant difference among group delay differences at different sound pressure levels (F(4,20) = [0.4969], *p* = 0.6945). The data was collected from five sensitive cochleae through the lateral wall opening (LWO) and presented by means and standard errors. (**b**), Means and standard errors of the group delay difference between the RL and BM vibration measured through the LWO and the round window (RW). The group delay difference measured in the transverse direction through the LWO is significantly larger than that measured through the RW (t = 10.4798, *p* < 0.0001).

The group delay difference was derived from the slope of the linear regression line of the phase difference curve and is presented in Fig. 4a. The data was collected in five sensitive cochleae at sound pressure levels of 50–80 dB SPL at 10 dB per step. The mean of group delay differences ranges from 23.7 to 26.2 μs. A one-way ANOVA revealed that there was no statistically significant difference among different sound pressure levels (F = (4, 20) = [0.4969], *p* = 0.6945). The lack of level-dependent change allows calculating the mean of the group delay across sound pressure levels. The mean of the group delay difference between the RL and BM vibration measured in the transverse direction through the lateral wall opening (LWO) is 25.1 ± 0.6 μs (n = 5), which is significantly larger than that measured through the round window (RW) (18.3 ± 0.2 μs, n = 7) (Fig. 4b)^22^ (t = 10.4798, *p* < 0.0001).

## Discussion

Most of our knowledge on the cochlear active process, or amplification, is based on the observation of the high sensitivity, sharp tuning, and nonlinear compression of the cochlear partition vibrations^31^. These features of the normal cochlear mechanical responses were revealed mainly by the vibration measurements from the basal turn of the cochlea. There are two approaches for accessing the cochlear partition, one is through an opening in the lateral wall of the basal cochlear turn^32–38^, and the other is through the round window^30,39,40^. The former approach allows measuring the vibration approximately in the transverse direction. The data collected in different experimental animals using this approach demonstrate that the auditory system's exceptional frequency selectivity and huge dynamic range are achieved mechanically in the cochlea^31,41^. Since the stapedial artery in the lateral wall of the basal cochlear turn blocks the optical access to the cochlear partition in gerbils, the BM vibration has been measured through the round window in this commonly used animal species^7,8,18,19,30,40^. The spatial relationship between the round window and the cochlear partition determined by the cochlear anatomy prevents the object beam of the interferometer from accessing the cochlear partition in the transverse direction (Fig. 1a). Despite this restriction, the cochlear partition vibrations measured through the round window in gerbils show the same tuning and nonlinearity as those measured in the transverse direction through the opening in the lateral wall in other experimental animals^31^.

Micromechanical measurements through the round window in gerbils demonstrate that the vibrations of the RL and the outer hair cell region are more robust than the BM vibration and physiologically vulnerable at the best frequency and frequencies far below the best frequency^7,8,18,22,23^. Moreover, heterodyne low-coherence interferometry through the round window reveals the phase difference between the RL and BM vibration^22^. The phase difference indicates that the RL vibration lags the BM vibration. The RL and BM move approximately in opposite directions at the basal part and in the same direction at the peak region of the traveling wave. These new micromechanical data indicate a global hydromechanical mechanism for cochlear amplification^24,26,42^.

Considering the complex three-dimensional structures of the cochlear partition, interpretation of relative motions within the cochlear partition requires more caution than that of single-location data. The phase difference between the RL and BM vibration has been interpreted as a result of the measurement error caused by a non-perpendicular optical axis. When the optical axis is not in the transverse direction of the cochlear partition, the object beam accesses the RL and BM at different longitudinal locations (indicated by locations A and C in Fig. 1b). The longitudinal location difference can result in a phase difference between the RL and BM vibration^27^. This is a legitimate concern because the optical axis might not be precisely in the transverse direction despite a great effort to optimize the access angle of the object beam in the recent experiments^23,28,43^.

**Figure 5:**
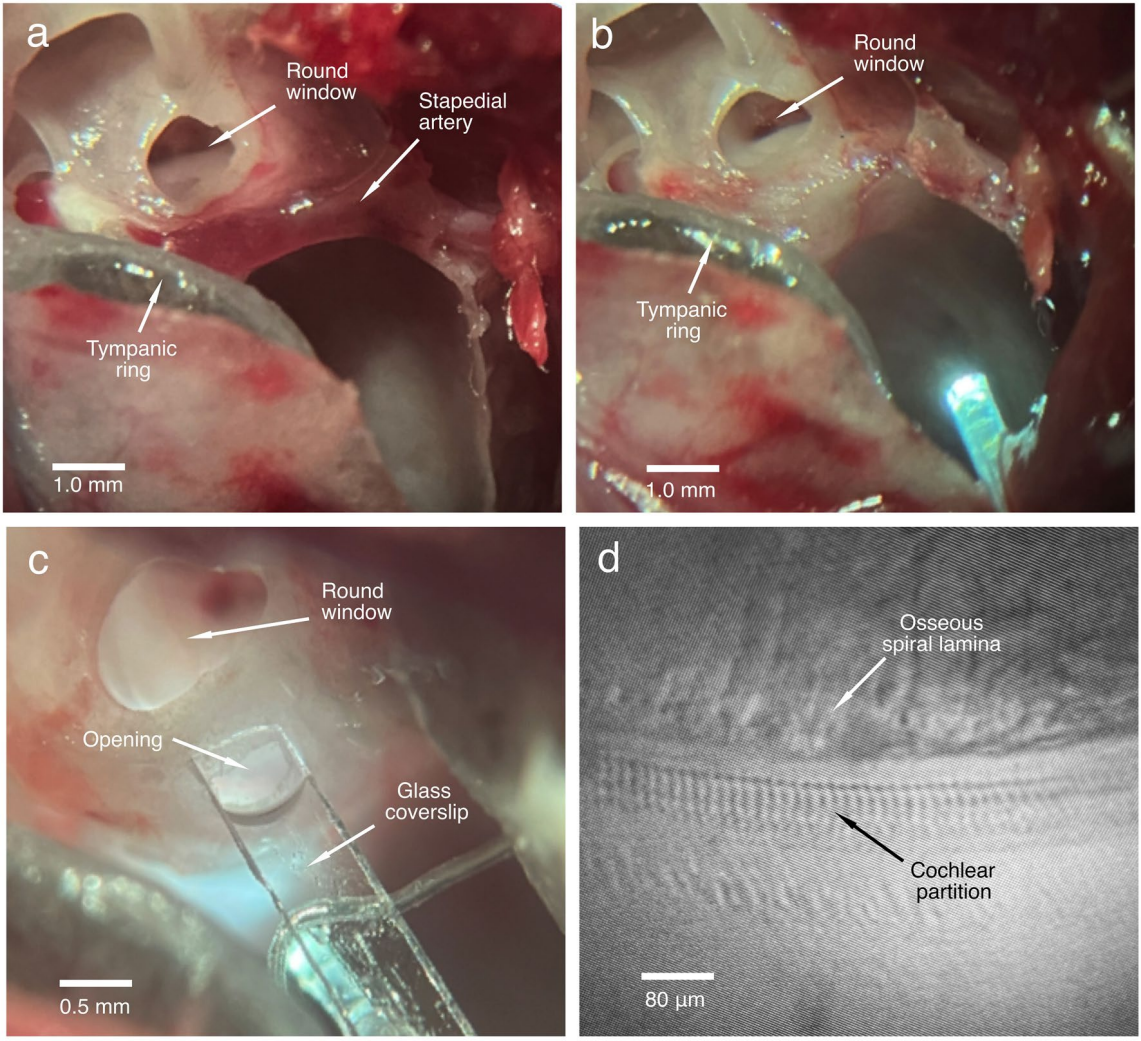
Images of the surgical field and the cochlear partition. (**a**), The stapedial artery blocks the optical access to the cochlear partition in the transverse direction. (**b**), The stapedial artery and surrounding bony structure have been removed from the basal turn. (**c**), An opening was made in the bony lateral wall of the scala tympani to visualize the cochlear partition. (**d**), Micrograph of the osseous spiral lamina and the cochlear partition in a sensitive cochlea. Images (**a**–**c**) were taken through a surgical microscope and (**d**) was a screen snapshot through a custom microscope using an objective lens (Plan Apo 20X, NA 0.28, Mitutoyo, Japan).

The objective of the present study was to determine whether the observed phase difference between the RL and BM vibration is a measurement error by measuring cochlear partition vibration in the transverse direction through an opening in the lateral wall of the scala tympani. Compared with the round window approach, the shorter distance between the lateral wall opening and the cochlear partition allows the object beam of the interferometer to access the partition approximately in the transverse direction. This is indicated by the image of the cochlear partition at the measured longitudinal location (Fig. 5d). If the phase difference results from the measurement error due to the extra delay from the longitudinal location B to C caused by the non-perpendicular optical axis (Fig. 1b), the group delay difference between the RL and BM vibration should become smaller when it is measured in the transverse direction. However, the present data show that the group delay difference between the RL and BM vibration measured in the transverse direction through the LWO is significantly larger than that measured with a non-perpendicular optical axis through the RW^24,26,42^ (Fig. 4b). This result does not support the hypothesis that the non-perpendicular optical axis causes the group delay difference between the RL and BM vibration. The larger group delay difference measured in this study likely results from changes in the spatial relationship between the object beam and the microstructures of the cochlear partition. This interpretation, however, remains to be confirmed by measuring the three-dimensional vibration^44^ from different locations of the microstructures within the cochlear partition. Moreover, the predicted measurement error-induced phase difference between the RL and BM vibration decreases with frequency with an increased rate (Fig. S5 by Motallebzadeh et al.^27^), which is similar to the traveling wave phase pattern (Figs. 1g,h and 2e,f). In contrast to this prediction, the measured phase difference between the RL and BM vibration decreases with frequency with an approximately constant rate, which forms a linear phase pattern (Figs. 1j and 2h). Even if the assumed measurement error exists, a 40-μm distance between location B and location C (Fig. 1b) results in only less than 3-μs delay considering the average speed of traveling wave at the base of the gerbil cochlea (~ 15 m/s)^45^, which is significantly smaller than the measured group delay difference between the RL and BM vibration (Fig. 4). Thus, the group delay difference between the RL and BM vibration cannot be interpreted as a measurement error.

The stapedial artery passing through the bony lateral wall makes it difficult to measure the cochlear partition vibration through the lateral wall of the basal turn in gerbil cochleae. This difficulty was overcome by developing a microsurgical procedure in this study. While it remains a complex procedure, the sensitive data collected in the current study demonstrates the feasibility of this new approach. Since the gerbils have been commonly used for cochlear mechanical study, the reported method is useful not only for heterodyne low-coherence interferometry but also for optical coherence tomography and for imaging the organ of Corti in vivo^46^.

In summary, a new surgical approach was developed for measuring the RL and BM vibrations in the transverse direction at the basal cochlear turn in gerbils. The present result demonstrates that the latency of the RL vibration is larger than that of BM vibration, which is consistent with the results measured through the round window. Confirmation of the time difference between the RL and BM vibration is imperative because the phase data plays an essential role in understanding how different microstructures of the cochlear partition work together for the cochlea amplification.

## Methods

### Animal preparation

Nineteen male and female young Mongolian gerbils with normal hearing at the age of 4 to 8 weeks and bodyweight of 40–80 g were used in this study. The experimental protocol was approved by the Oregon Health & Science University Institutional Animal Care and Use Committee (Protocol Number: IP00000932). All experiments were performed in accordance with relevant guidelines and regulations. The study is reported in accordance with ARRIVE guidelines.

The animal surgery and vibration measurement were performed on a vibration isolation table inside an acoustically attenuated double-wall booth. Anesthesia was induced by ketamine and xylazine (100 mg per kg and 10 mg per kg intramuscularly). A tracheotomy was performed to keep natural free breathing. Body temperature was kept constant at ~ 38 °C using a DC-powered heating blanket, which was feedback-controlled through a rectal temperature probe. The animal's head was cemented to a custom-built head holder mounted on a three-dimensional translational stage. Two speakers and one microphone were coupled to the ear canal through a speculum to form a closed sound field. Two continuous tones at 20 and 24 kHz at 60 dB SPL were used to evoke distortion product otoacoustic emission at 16 kHz. The emission measured from the ear canal was displayed on a signal analyzer (SR785, Stanford Research Systems, Sunnyvale, CA) and digitized using a lock-in amplifier (SR830 DSP, Stanford Research Systems, Sunnyvale, CA). A cochlea with a < 6-dB decrease of the otoacoustic emission was considered a sensitive preparation.

### Surgeries for accessing the cochlear partition in the transverse direction

The auditory bulla on the left side was exposed through a ventrolateral surgical approach. The anterior and lateral bony walls of the bulla were carefully removed using a sharp blade to visualize the basal turn of the cochlea and the stapedial artery^28,29,47^ (Fig. 5a). Under a surgical microscope, the ventral wall of the bony canal encircling the stapedial artery was gently shaved away using a small sharp blade. After the vessel was separated from the bony surface, it was ligated at two locations, one at a medial location close to the carotid artery and the other at a distal location at the edge of the round window. The stapedial artery was transected between the two knots, and the remaining bony structure underneath the stapedial artery was removed from the surface of the lateral wall of the basal cochlear turn (Fig. 5b). An opening of ~ 0.3 mm x ~ 0.5 mm was made in the lateral wall of the scala tympani at the ~ 20-kHz location by shaving off the bony wall (Fig. 5c). The animal's head was turned into a position so that the bone chips would not fall onto the BM. A glass coverslip was placed on the opening to avoid the optical distortion caused by the curved fluid surface. Due to the surface tension, perilymph usually filled the space between the opening and the glass coverslip in a few minutes. A light beam through a single-mode optical fiber was brought close to the lateral bony wall of the scalae media and vestibuli to illuminate the cochlear partition. The position and orientation of the optical fiber were adjusted so that landmarks of the cochlear partition were visible. The cochlear partition at the measured longitudinal location was positioned approximately in the horizontal plane, confirmed by the image through the objective lens (Fig. 5d).

### Measurement of the reticular lamina and basilar membrane vibrations

For the vibration measurement, the angle between the glass coverslip and the cochlear partition was adjusted to allow the object beam to access the RL and BM in the transverse direction confirmed by the image through a long working distance objective lens (Plan Apo 20X, NA 0.28, Mitutoyo, Japan)^43^. A custom-built heterodyne low-coherence interferometer was used to measure the cochlear partition vibrations^2^. When the object beam from the interferometer was focused on the center of the outer hair cell region, the vibration magnitude and phase in response to a 20-kHz tone at an intermediate sound pressure level, and carrier signal, i.e., the backscattered light level, were measured as a function of the transverse location. The transverse locations of the BM and RL were determined by the peaks of the carrier signal^22,43^. When the object beam of the interferometer was focused on those locations sequentially, the vibrations were measured at different frequencies and sound pressure levels.

A digital lock-in amplifier (SR830 DSP, Stanford Research Systems, Sunnyvale, CA) was used for signal generation and data acquisition. A continuous sinusoidal signal produced by the lock-in amplifier was used to drive one of the two electrostatic speakers (EC1, Tucker-Davis Technologies, Alachua, FL) through a power amplifier. The sound pressure level in the ear canal was controlled by changing the signal level to the amplifier using an attenuator (PA5, Tucker-Davis Technologies, Alachua, FL). The tone frequency varied from 0.75 to 33 kHz by 0.75 kHz per step, and the sound pressure level was changed from 20 to 80 dB SPL by 10 dB per step. At given sound pressure levels and frequencies, the vibration magnitude and phase were measured by the lock-in amplifier and stored in a computer. The carrier signal level was monitored for detecting and rejecting the heartbeat- and respiration-induced movement artifacts. When the carrier signal was low, indicating a poor signal-to-noise ratio, the vibration magnitude and phase were discarded. The stapes vibrations were also measured to determine the transfer functions of the cochlear partition vibrations.

### Statistics and reproducibility

Igor Pro (Version 8.04, WaveMetrics, Lake Oswego, OR) was used for data processing and statistical analysis. The frequency responses of the RL and BM vibrations were presented by the displacement as a function of frequency (Figs. 1c,d, 2a,b). The sensitivities of the RL and BM vibrations were presented by the ratios of the RL or BM displacement to the stapes displacement as a function of frequency (Figs. 1e,f, 2c,d). The phase responses of the cochlear partition were displayed by the phase difference obtained by subtracting the stapes phase from the RL or BM phase as a function of frequency (Figs. 1g,h, 2e,f). The magnitude difference between the RL and BM vibration was shown by the ratio of the RL vibration displacement to the BM displacement as a function of frequency (Figs. 1i and 2g). The phase difference between the RL and BM was calculated by subtracting the BM phase from the RL phase (Figs. 1j and 2h). The group delay difference $\tau$ was derived from the phase difference according to $\tau = - \Delta \varphi /2\pi \Delta f$, where $\tau$ is in second, $\Delta \varphi$ in radian, $\Delta f$ in hertz, and $\Delta \varphi /\Delta f$ was obtained through a linear regression based on the phase difference-frequency function. The group delay was calculated over the frequency range of 0.1–0.9 BF. This frequency range was chosen because the signal below 0.1 BF is too noisy, and the RL and BM response peaks are at frequencies below the BF at intermediate and high sound pressure levels. The group delay difference was also calculated from phase data measured through the round window in a previous study^22^. Except for the measurement-angle difference, the methods for measuring the RL and BM vibrations are the same in the present and previous experiments. The group delay differences at different sound pressure levels were analyzed using one-way ANOVA. The difference between the BM-RL group delay measured in the transverse direction and that measured through the round window was analyzed using a two-tailed t-test. A probability smaller than 0.05 was considered statistically significant.

## Data Availability

Data are available from the corresponding author.
